# Supplementary material for: Underlying Causes of Myocardial Infarction with Nonobstructive Coronary Arteries: Optical Coherence Tomography and Cardiac Magnetic Resonance Imaging Pilot Study
Source: J Clin Med. 2022 Dec 17;11(24):7495. doi: 10.3390/jcm11247495 (PMC9784746; doi:10.3390/jcm11247495)
Supplement: Supplementary file 1 [file jcm-11-07495-s001.zip › jcm-2076705-supplementary.pdf]

Supplement 1. Baseline characteristics.

Patient 1

**Age**	46
**Female**	No
**NSTEMI**	No
**Typical angina**	No
**LVEF (%)**	40
**RWMA**	Yes
**Severe valvular disease**	No
**Pericardial effusion**	No
**Heart Failure**	
**NYHA I**	No
**NYHA II**	Yes
**NYHA III**	No
**NYHA IV**	No
**Hypertension**	Yes
**Hypercholesterolaemia**	No
**Smoking**	Yes
**Diabetes**	No
**Obestiy**	No
**Previous CAD**	No
**Previous stroke**	No
**Previous kidney disease**	No
**Previous lung disease**	No
**Family history**	No
**Sinus rhythm in ECG**	Yes

NSTEMI = non-ST segment elevation myocardial infarction. HR = heart rate. LVEF = left ventricle ejection fraction. RWMA = regional wall motion abnormalities, CAD = coronary artery disease, PAD = peripheral artery disease

Patient 2

**Age**	41
**Female**	Yes
**NSTEMI**	Yes
**Typical angina**	Yes
**LVEF (%)**	70
**RWMA**	No
**Severe valvular disease**	No
**Pericardial effusion**	No
**Heart Failure**	
**NYHA I**	Yes
**NYHA II**	No
**NYHA III**	No
**NYHA IV**	No
**Hypertension**	No
**Hypercholesterolaemia**	No
**Smoking**	No
**Diabetes**	No
**Obestiy**	No
**Previous CAD**	No
**Previous stroke**	No
**Previous kidney disease**	No
**Previous lung disease**	No
**Family history**	No
**Sinus rhythm in ECG**	Yes

NSTEMI = non-ST segment elevation myocardial infarction. HR = heart rate. LVEF = left ventricle ejection fraction. RWMA = regional wall motion abnormalities, CAD = coronary artery disease, PAD = peripheral artery disease

Patient 3

**Age**	55
**Female**	No
**NSTEMI**	No
**Typical angina**	Yes
**LVEF (%)**	55
**RWMA**	No
**Severe valvular disease**	No
**Pericardial effusion**	No
**Heart Failure**	
**NYHA I**	No
**NYHA II**	Yes
**NYHA III**	No
**NYHA IV**	No
**Hypertension**	Yes
**Hypercholesterolaemia**	No
**Smoking**	No
**Diabetes**	No
**Obestiy**	No
**Previous CAD**	No
**Previous stroke**	No
**Previous kidney disease**	No
**Previous lung disease**	No
**Family history**	No
**Sinus rhythm in ECG**	Yes

NSTEMI = non-ST segment elevation myocardial infarction. HR = heart rate. LVEF = left ventricle ejection fraction. RWMA = regional wall motion abnormalities, CAD = coronary artery disease, PAD = peripheral artery disease

Patient 4

**Age**	64
**Female**	Yes
**NSTEMI**	Yes
**Typical angina**	Yes
**LVEF (%)**	60
**RWMA**	No
**Severe valvular disease**	No
**Pericardial effusion**	Yes
**Heart Failure**	
**NYHA I**	Yes
**NYHA II**	No
**NYHA III**	No
**NYHA IV**	No
**Hypertension**	Yes
**Hypercholesterolaemia**	Yes
**Smoking**	No
**Diabetes**	No
**Obestiy**	No
**Previous CAD**	No
**Previous stroke**	No
**Previous kidney disease**	No
**Previous lung disease**	No
**Family history**	Yes
**Sinus rhythm in ECG**	Yes

NSTEMI = non-ST segment elevation myocardial infarction. HR = heart rate. LVEF = left ventricle ejection fraction. RWMA = regional wall motion abnormalities, CAD = coronary artery disease, PAD = peripheral artery disease

Patient 5

**Age**	54
**Female**	No
**NSTEMI**	No
**Typical angina**	No
**LVEF (%)**	60
**RWMA**	No
**Severe valvular disease**	No
**Pericardial effusion**	No
**Heart Failure**	
**NYHA I**	Yes
**NYHA II**	No
**NYHA III**	No
**NYHA IV**	No
**Hypertension**	Yes
**Hypercholesterolaemia**	No
**Smoking**	Yes
**Diabetes**	No
**Obestiy**	No
**Previous CAD**	No
**Previous stroke**	No
**Previous kidney disease**	No
**Previous lung disease**	No
**Family history**	No
**Sinus rhythm in ECG**	Yes

NSTEMI = non-ST segment elevation myocardial infarction. HR = heart rate. LVEF = left ventricle ejection fraction. RWMA = regional wall motion abnormalities, CAD = coronary artery disease, PAD = peripheral artery disease

Patient 6

**Age**	56
**Female**	Yes
**NSTEMI**	Yes
**Typical angina**	Yes
**LVEF (%)**	50
**RWMA**	Yes
**Severe valvular disease**	No
**Pericardial effusion**	No
**Heart Failure**	
**NYHA I**	Yes
**NYHA II**	No
**NYHA III**	No
**NYHA IV**	No
**Hypertension**	No
**Hypercholesterolaemia**	No
**Smoking**	Yes
**Diabetes**	No
**Obestiy**	No
**Previous CAD**	No
**Previous stroke**	No
**Previous kidney disease**	No
**Previous lung disease**	No
**Family history**	No
**Sinus rhythm in ECG**	Yes

NSTEMI = non-ST segment elevation myocardial infarction. HR = heart rate. LVEF = left ventricle ejection fraction. RWMA = regional wall motion abnormalities, CAD = coronary artery disease, PAD = peripheral artery disease

Patient 7

**Age**	49
**Female**	No
**NSTEMI**	Yes
**Typical angina**	Yes
**LVEF**	30
**RWMA**	Yes
**Severe valvular disease**	No
**Pericardial effusion**	No
**Heart Failure**	
**NYHA I**	No
**NYHA II**	Yes
**NYHA III**	No
**NYHA IV**	No
**Hypertension**	Yes
**Hypercholesterolaemia**	No
**Smoking**	Yes
**Diabetes**	No
**Obestiy**	No
**Previous CAD**	No
**Previous stroke**	No
**Previous kidney disease**	No
**Previous lung disease**	No
**Family history**	No
**Sinus rhythm in ECG**	Yes

NSTEMI = non-ST segment elevation myocardial infarction. HR = heart rate. LVEF = left ventricle ejection fraction. RWMA = regional wall motion abnormalities, CAD = coronary artery disease, PAD = peripheral artery disease

Patient 8

**Age**	49
**Female**	No
**NSTEMI**	Yes
**Typical angina**	No
**LVEF (%)**	55
**RWMA**	No
**Severe valvular disease**	No
**Pericardial effusion**	No
**Heart Failure**	
**NYHA I**	Yes
**NYHA II**	No
**NYHA III**	No
**NYHA IV**	No
**Hypertension**	Yes
**Hypercholesterolaemia**	No
**Smoking**	Yes
**Diabetes**	No
**Obestiy**	Yes
**Previous CAD**	No
**Previous stroke**	No
**Previous kidney disease**	No
**Previous lung disease**	No
**Family history**	Yes
**Sinus rhythm in ECG**	Yes

NSTEMI = non-ST segment elevation myocardial infarction. HR = heart rate. LVEF = left ventricle ejection fraction. RWMA = regional wall motion abnormalities, CAD = coronary artery disease, PAD = peripheral artery disease

Patient 9

**Age**	52
**Female**	Yes
**NSTEMI**	Yes
**Typical angina**	Yes
**LVEF (%)**	30
**RWMA**	Yes
**Severe valvular disease**	No
**Pericardial effusion**	No
**Heart Failure**	
**NYHA I**	No
**NYHA II**	Yes
**NYHA III**	No
**NYHA IV**	No
**Hypertension**	Yes
**Hypercholesterolaemia**	No
**Smoking**	Yes
**Diabetes**	No
**Obestiy**	No
**Previous CAD**	No
**Previous stroke**	No
**Previous kidney disease**	No
**Previous lung disease**	No
**Family history**	Yes
**Sinus rhythm in ECG**	Yes

NSTEMI = non-ST segment elevation myocardial infarction. HR = heart rate. LVEF = left ventricle ejection fraction. RWMA = regional wall motion abnormalities, CAD = coronary artery disease, PAD = peripheral artery disease

Patient 10

**Age**	55
**Female**	Yes
**NSTEMI**	Yes
**Typical angina**	Yes
**LVEF (%)**	60
**RWMA**	No
**Severe valvular disease**	No
**Pericardial effusion**	No
**Heart Failure**	
**NYHA I**	Yes
**NYHA II**	No
**NYHA III**	No
**NYHA IV**	No
**Hypertension**	Yes
**Hypercholesterolaemia**	Yes
**Smoking**	Yes
**Diabetes**	Yes
**Obestiy**	No
**Previous CAD**	No
**Previous stroke**	No
**Previous kidney disease**	No
**Previous lung disease**	No
**Family history**	No
**Sinus rhythm in ECG**	Yes

NSTEMI = non-ST segment elevation myocardial infarction. HR = heart rate. LVEF = left ventricle ejection fraction. RWMA = regional wall motion abnormalities, CAD = coronary artery disease, PAD = peripheral artery disease

Supplement 2. OCT and CMR findings.

Patient 1

**OCT**	
**Plaque rupture**	No
**Plaque erosion**	No
**Eruptive calcific nodule**	No
**Spontaneous coronary artery dissection**	No
**Presence of thrombus**	No
**Negative OCT**	Yes
**CMR**	
**T2 hyperintensity**	
**Present**	Yes
**Absent**	No
**Myocardial hemorrhage**	No
**MVO**	No
**Abnormal delayed enhancement**	
**Subendocardial**	No
**Transmural**	Yes
**Subendocardial + transmural**	No
**LV dimensions and function**	
**LVEDV (ml/m^2)**	171
**LVESV (ml/m^2)**	80
**LVEF (%)**	53
**Pericardial effusion**	No
**Definite diagnosis**	
**MI**	No
**Myocarditis**	Yes
**Negative CMR**	No

OCT – optical coherence tomography, CMR – cardiac magnetic resonance, MVO – microvascular obstruction, LV – left ventricle, MI – myocardial infarction.

Patient 2

**OCT**	
**Plaque rupture**	Yes
**Plaque erosion**	No
**Eruptive calcific nodule**	No
**Spontaneous coronary artery dissection**	Yes
**Presence of thrombus**	No
**Negative OCT**	No
**CMR**	
**T2 hyperintensity**	
**Present**	Yes
**Absent**	No
**Myocardial hemorrhage**	No
**MVO**	No
**Abnormal delayed enhancement**	
**Subendocardial**	Yes
**Transmural**	No
**Subendocardial + transmural**	No
**LV dimensions and function**	
**LVEDV (ml/m^2)**	105
**LVESV (ml/m^2)**	42
**LVEF (%)**	60
**Pericardial effusion**	No
**Definite diagnosis**	
**MI**	Yes
**Myocarditis**	No
**Negative CMR**	No

OCT – optical coherence tomography, CMR – cardiac magnetic resonance, MVO – microvascular obstruction, LV – left ventricle, MI – myocardial infarction.

Patient 3

**OCT**	
**Plaque rupture**	No
**Plaque erosion**	Yes
**Eruptive calcific nodule**	No
**Spontaneous coronary artery dissection**	No
**Presence of thrombus**	Yes
**Negative OCT**	No
**CMR**	
**T2 hyperintensity**	
**Present**	Yes
**Absent**	No
**Myocardial hemorrhage**	No
**MVO**	No
**Abnormal delayed enhancement**	
**Subendocardial**	Yes
**Transmural**	Yes
**Subendocardial + transmural**	Yes
**LV dimensions and function**	
**LVEDV (ml/m^2)**	127
**LVESV (ml/m^2)**	28
**LVEF (%)**	78
**Pericardial effusion**	No
**Definite diagnosis**	
**MI**	Yes
**Myocarditis**	No
**Negative CMR**	No

OCT – optical coherence tomography, CMR – cardiac magnetic resonance, MVO – microvascular obstruction, LV – left ventricle, MI – myocardial infarction.

Patient 4

**OCT**	
**Plaque rupture**	No
**Plaque erosion**	Yes
**Eruptive calcific nodule**	No
**Spontaneous coronary artery dissection**	No
**Presence of thrombus**	No
**Negative OCT**	No
**CMR**	
**T2 hyperintensity**	
**Present**	Yes
**Absent**	No
**Myocardial hemorrhage**	No
**MVO**	No
**Abnormal delayed enhancement**	
**Subendocardial**	Yes
**Transmural**	Yes
**Subendocardial + transmural**	Yes
**LV dimensions and function**	
**LVEDV (ml/m^2)**	94
**LVESV (ml/m^2)**	33
**LVEF (%)**	65
**Pericardial effusion**	Yes
**Definite diagnosis**	
**MI**	Yes
**Myocarditis**	No
**Negative CMR**	No

OCT – optical coherence tomography, CMR – cardiac magnetic resonance, MVO – microvascular obstruction, LV – left ventricle, MI – myocardial infarction.

Patient 5

**OCT**	
**Plaque rupture**	No
**Plaque erosion**	No
**Eruptive calcific nodule**	No
**Spontaneous coronary artery dissection**	No
**Presence of thrombus**	Yes
**Negative OCT**	No
**CMR**	
**T2 hyperintensity**	
**Present**	Yes
**Absent**	No
**Myocardial hemorrhage**	No
**MVO**	No
**Abnormal delayed enhancement**	
**Subendocardial**	Yes
**Transmural**	Yes
**Subendocardial + transmural**	Yes
**LV dimensions and function**	
**LVEDV (ml/m^2)**	138
**LVESV (ml/m^2)**	45
**LVEF (%)**	68
**Pericardial effusion**	No
**Definite diagnosis**	
**MI**	Yes
**Myocarditis**	No
**Negative CMR**	No

OCT – optical coherence tomography, CMR – cardiac magnetic resonance, MVO – microvascular obstruction, LV – left ventricle, MI – myocardial infarction.

Patient 6

**OCT**	
**Plaque rupture**	No
**Plaque erosion**	No
**Eruptive calcific nodule**	No
**Spontaneous coronary artery dissection**	No
**Presence of thrombus**	No
**Negative OCT**	Yes
**CMR**	
**T2 hyperintensity**	
**Present**	Yes
**Absent**	No
**Myocardial hemorrhage**	No
**MVO**	No
**Abnormal delayed enhancement**	
**Subendocardial**	No
**Transmural**	Yes
**Subendocardial + transmural**	No
**LV dimensions and function**	
**LVEDV (ml/m^2)**	100
**LVESV (ml/m^2)**	41
**LVEF (%)**	59
**Pericardial effusion**	No
**Definite diagnosis**	
**MI**	No
**Myocarditis**	Yes
**Negative CMR**	No

OCT – optical coherence tomography, CMR – cardiac magnetic resonance, MVO – microvascular obstruction, LV – left ventricle, MI – myocardial infarction.

Patient 7

**OCT**	
**Plaque rupture**	No
**Plaque erosion**	No
**Eruptive calcific nodule**	No
**Spontaneous coronary artery dissection**	Yes
**Presence of thrombus**	No
**Negative OCT**	No
**CMR**	
**T2 hyperintensity**	
**Present**	No
**Absent**	Yes
**Myocardial hemorrhage**	No
**MVO**	No
**Abnormal delayed enhancement**	
**Subendocardial**	No
**Transmural**	No
**Subendocardial + transmural**	No
**LV dimensions and function**	
**LVEDV (ml/m^2)**	131
**LVESV (ml/m^2)**	63
**LVEF (%)**	52
**Pericardial effusion**	No
**Definite diagnosis**	
**MI**	Yes
**Myocarditis**	No
**Negative CMR**	No

OCT – optical coherence tomography, CMR – cardiac magnetic resonance, MVO – microvascular obstruction, LV – left ventricle, MI – myocardial infarction.

Patient 8

**OCT**	
**Plaque rupture**	Yes
**Plaque erosion**	No
**Eruptive calcific nodule**	No
**Spontaneous coronary artery dissection**	No
**Presence of thrombus**	No
**Negative OCT**	No
**CMR**	
**T2 hyperintensity**	
**Present**	Yes
**Absent**	No
**Myocardial hemorrhage**	No
**MVO**	Yes
**Abnormal delayed enhancement**	
**Subendocardial**	Yes
**Transmural**	Yes
**Subendocardial + transmural**	Yes
**LV dimensions and function**	
**LVEDV (ml/m^2)**	154
**LVESV (ml/m^2)**	63
**LVEF (%)**	59
**Pericardial effusion**	No
**Definite diagnosis**	
**MI**	Yes
**Myocarditis**	No
**Negative CMR**	No

OCT – optical coherence tomography, CMR – cardiac magnetic resonance, MVO – microvascular obstruction, LV – left ventricle, MI – myocardial infarction.

Patient 9

**OCT**	
**Plaque rupture**	No
**Plaque erosion**	No
**Eruptive calcific nodule**	No
**Spontaneous coronary artery dissection**	No
**Presence of thrombus**	No
**Negative OCT**	Yes
**CMR**	
**T2 hyperintensity**	
**Present**	Yes
**Absent**	No
**Myocardial hemorrhage**	No
**MVO**	No
**Abnormal delayed enhancement**	
**Subendocardial**	No
**Transmural**	No
**Subendocardial + transmural**	No
**LV dimensions and function**	
**LVEDV (ml/m^2)**	131
**LVESV (ml/m^2)**	63
**LVEF (%)**	52
**Pericardial effusion**	Yes
**Definite diagnosis**	
**MI**	No
**Myocarditis**	No
**Negative CMR**	No

OCT – optical coherence tomography, CMR – cardiac magnetic resonance, MVO – microvascular obstruction, LV – left ventricle, MI – myocardial infarction.

Patient 10

OCT	
**Plaque rupture**	No
**Plaque erosion**	No
**Eruptive calcific nodule**	No
**Spontaneous coronary artery dissection**	No
**Presence of thrombus**	Yes
**Negative OCT**	No
**CMR**	
**T2 hyperintensity**	
**Present**	Yes
**Absent**	No
**Myocardial hemorrhage**	No
**MVO**	No
**Abnormal delayed enhancement**	
**Subendocardial**	No
**Transmural**	Yes
**Subendocardial + transmural**	No
**LV dimensions and function**	
**LVEDV (ml/m^2)**	100
**LVESV (ml/m^2)**	41
**LVEF (%)**	59
**Pericardial effusion**	No
**Definite diagnosis**	
**MI**	Yes
**Myocarditis**	No
**Negative CMR**	No

OCT – optical coherence tomography, CMR – cardiac magnetic resonance, MVO – microvascular obstruction, LV – left ventricle, MI – myocardial infarction.
